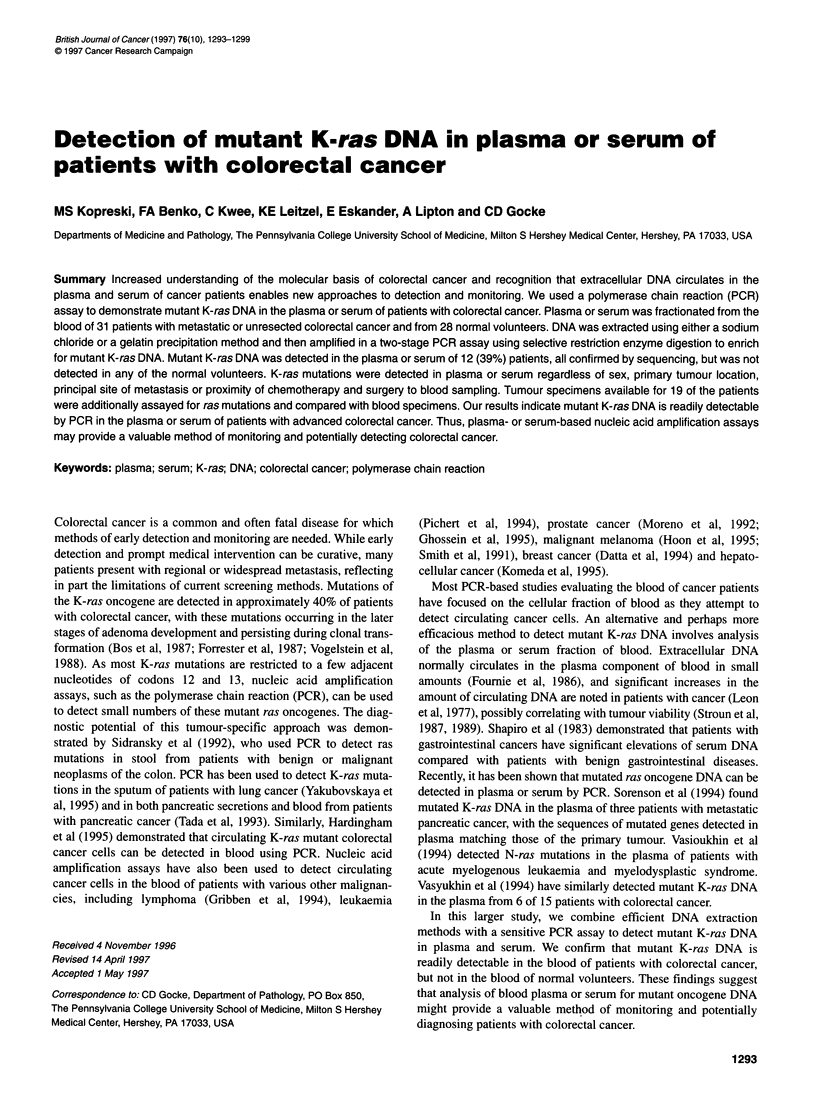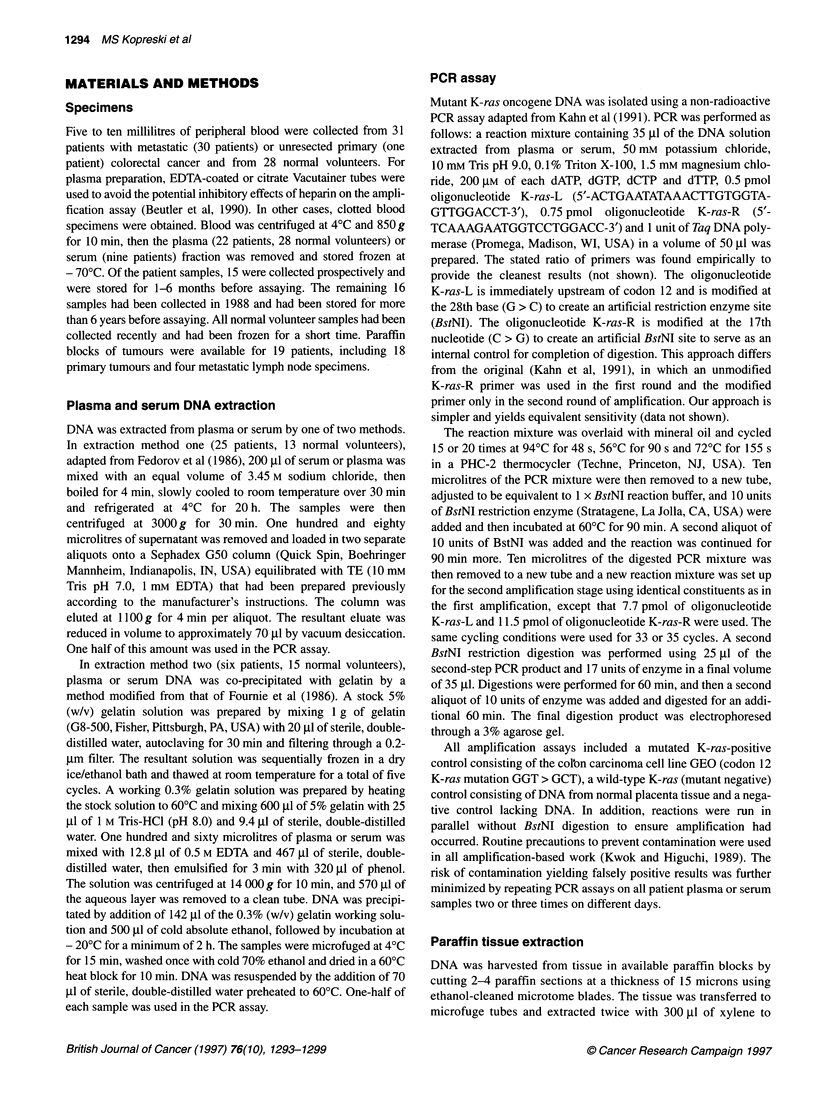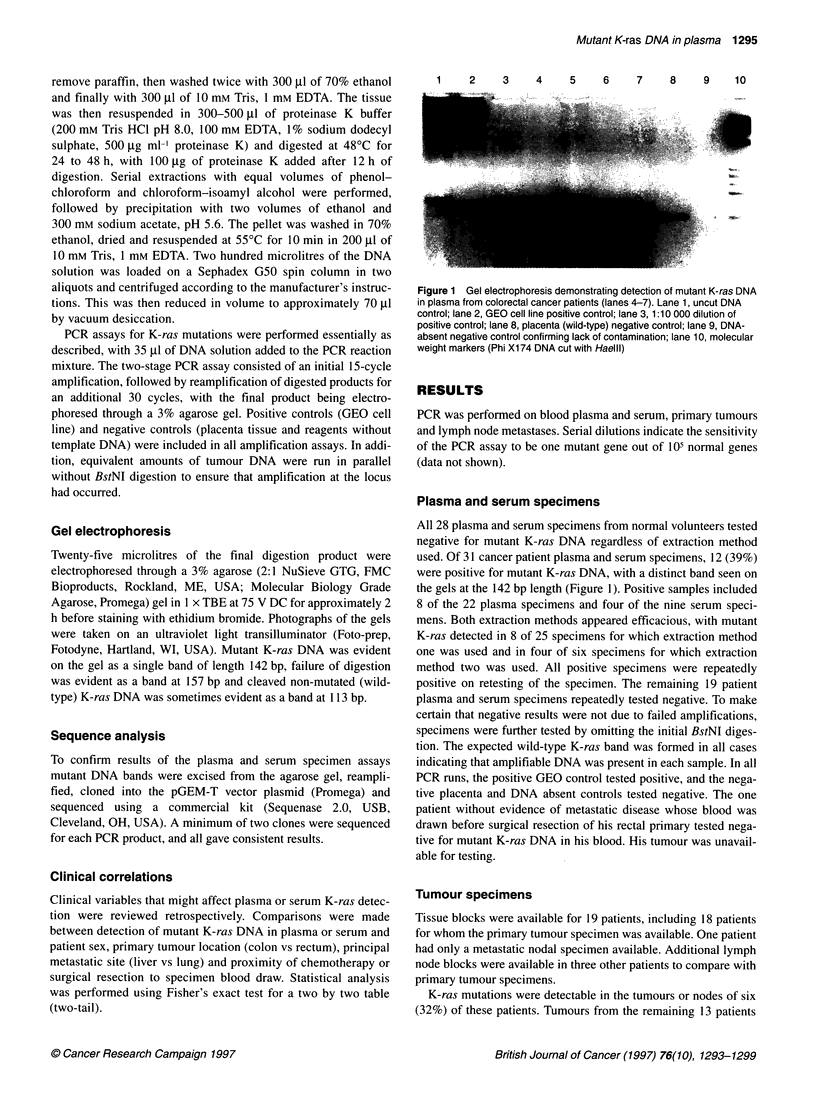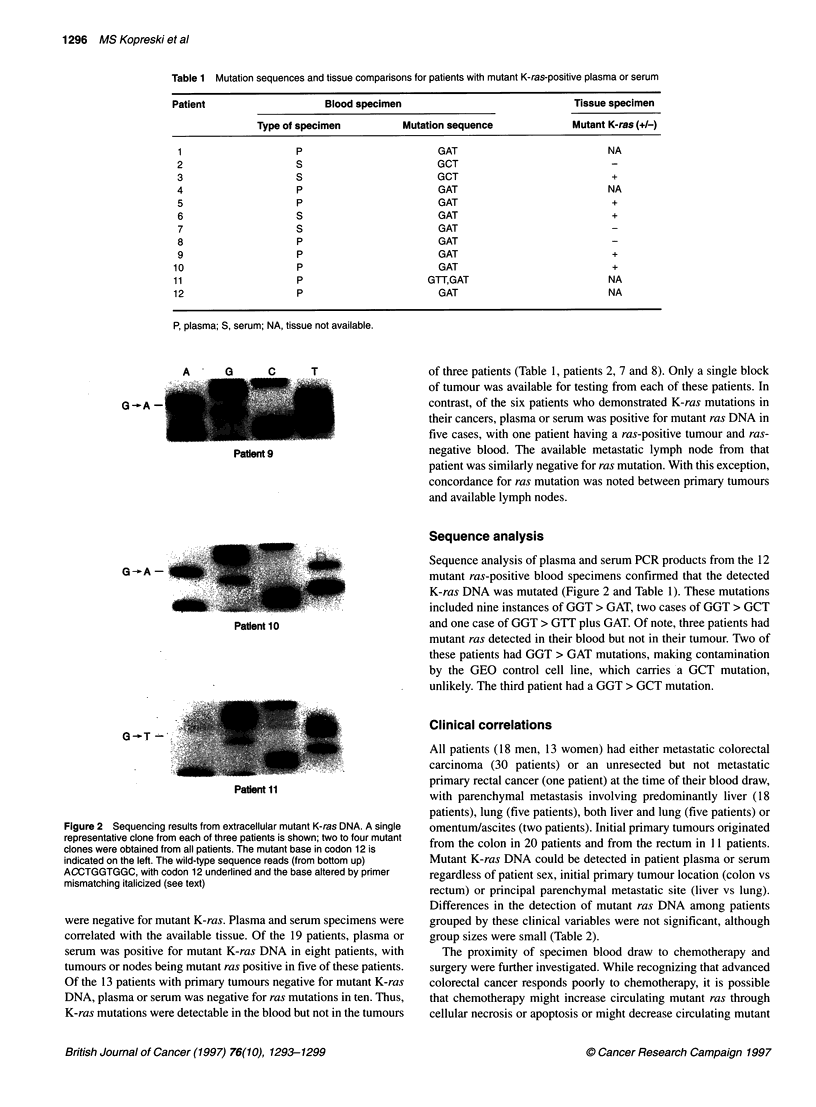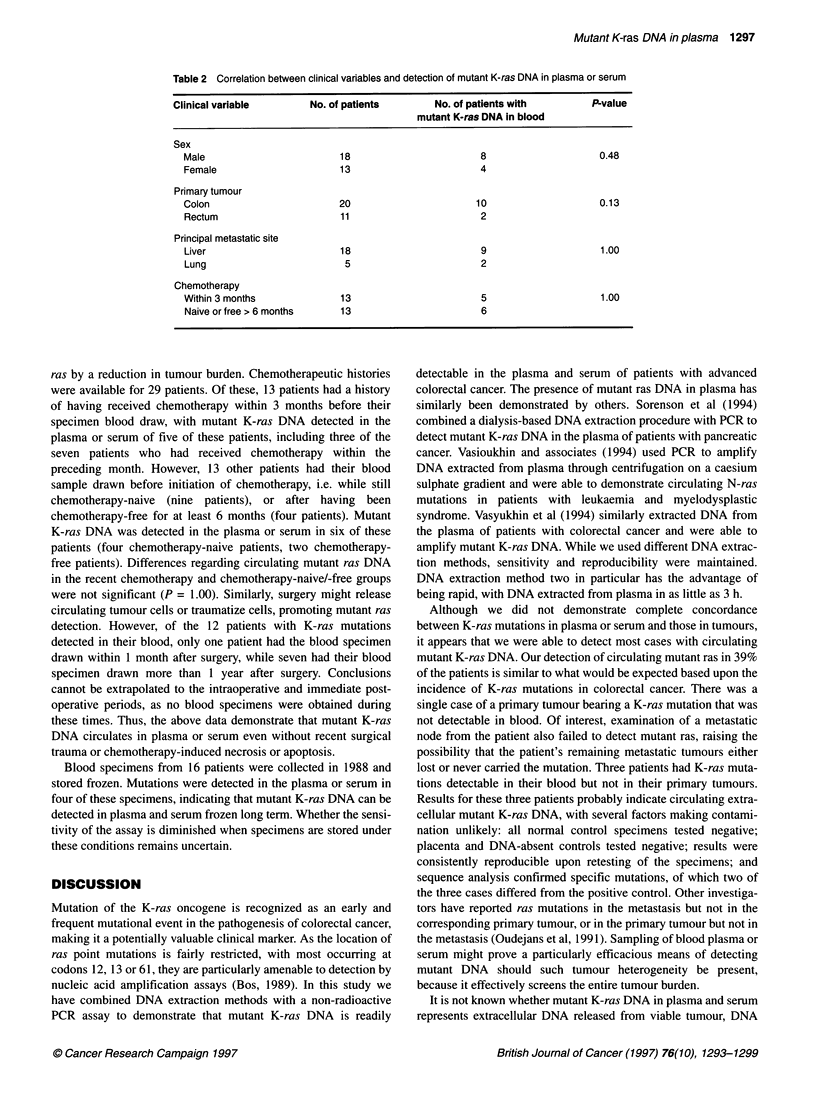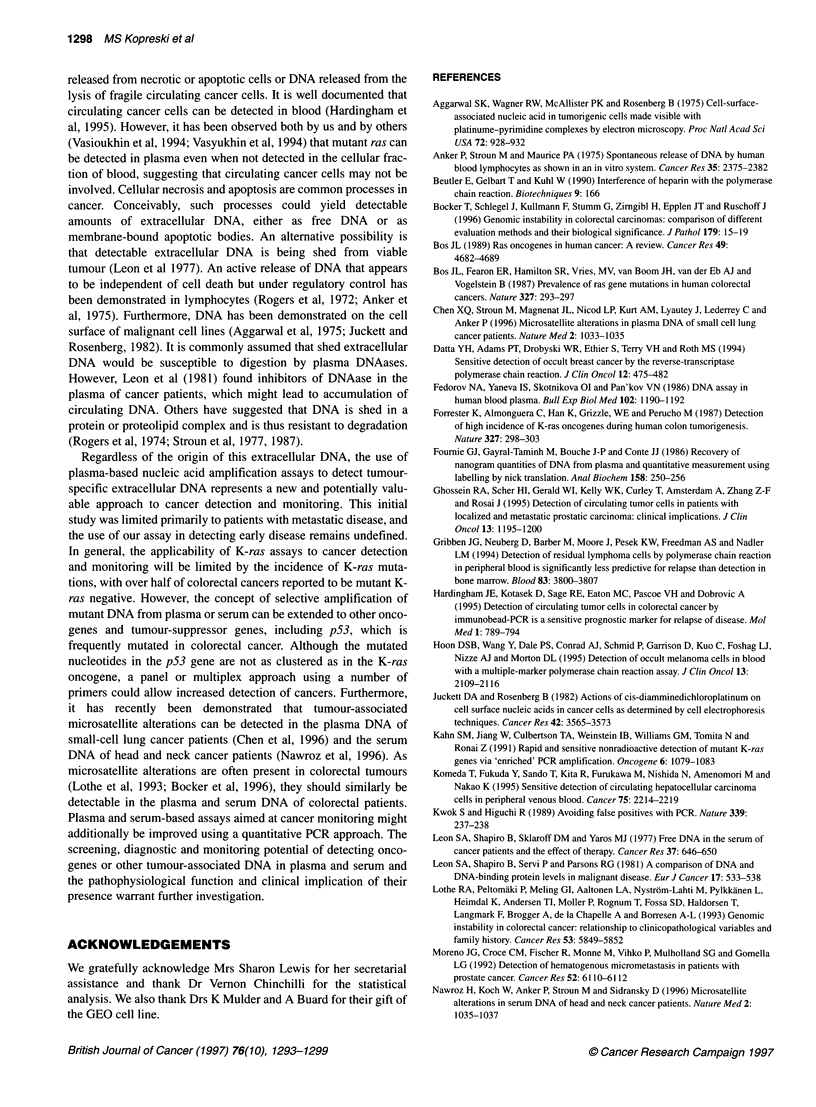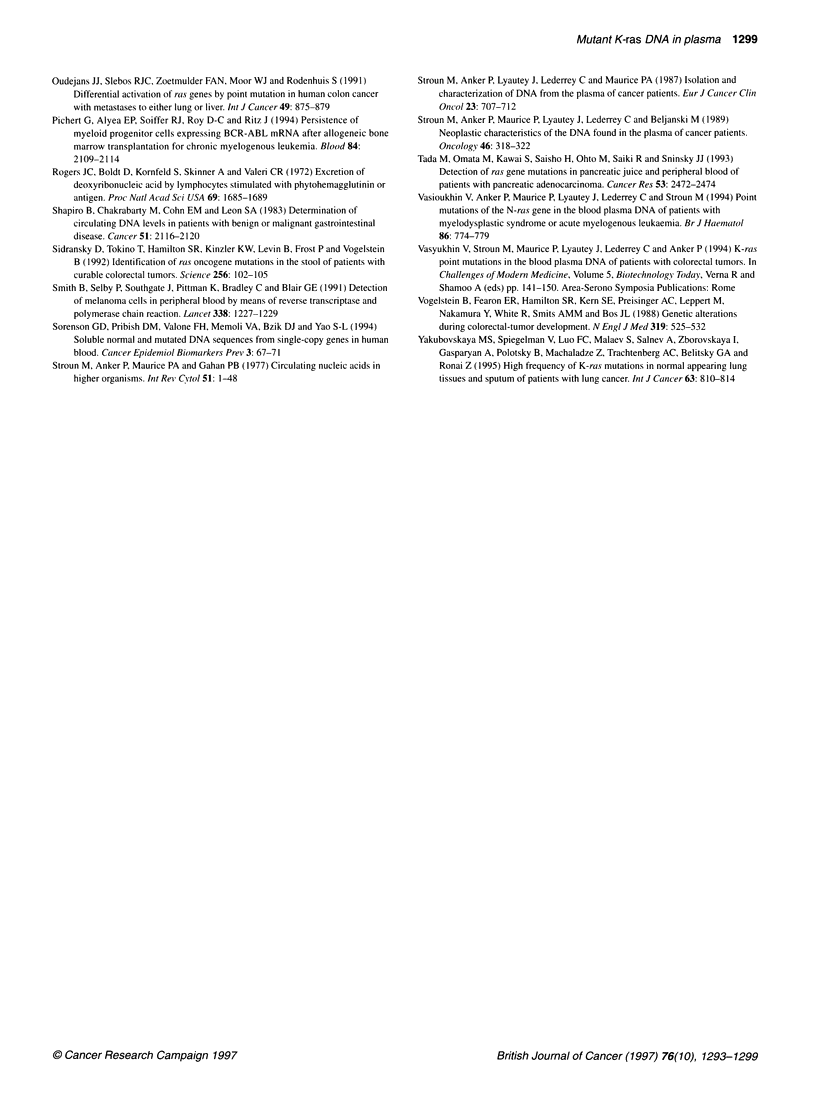# Detection of mutant K-ras DNA in plasma or serum of patients with colorectal cancer.

**DOI:** 10.1038/bjc.1997.551

**Published:** 1997

**Authors:** M. S. Kopreski, F. A. Benko, C. Kwee, K. E. Leitzel, E. Eskander, A. Lipton, C. D. Gocke

**Affiliations:** Department of Medicine, The Pennsylvania College University School of Medicine, Milton S Hershey Medical Center, Hershey, PA 17033, USA.

## Abstract

**Images:**


British Journal of Cancer (1997) 76(10), 1293–1299
© 1997 Cancer Research Campaign

### Detection of mutant K-*ras* DNA in plasma or serum of patients with colorectal cancer

**MS Kopreski, FA Benko, C Kwee, KE Leitzel, E Eskander, A Lipton and CD Gocke**

Departments of Medicine and Pathology, The Pennsylvania College University School of Medicine, Milton S Hershey Medical Center, Hershey, PA 17033, USA

**Summary** Increased understanding of the molecular basis of colorectal cancer and recognition that extracellular DNA circulates in the plasma and serum of cancer patients enables new approaches to detection and monitoring. We used a polymerase chain reaction (PCR) assay to demonstrate mutant K-*ras* DNA in the plasma or serum of patients with colorectal cancer. Plasma or serum was fractionated from the blood of 31 patients with metastatic or unresected colorectal cancer and from 28 normal volunteers. DNA was extracted using either a sodium chloride or a gelatin precipitation method and then amplified in a two-stage PCR assay using selective restriction enzyme digestion to enrich for mutant K-*ras* DNA. Mutant K-*ras* DNA was detected in the plasma or serum of 12 (39%) patients, all confirmed by sequencing, but was not detected in any of the normal volunteers. K-*ras* mutations were detected in plasma or serum regardless of sex, primary tumour location, principal site of metastasis or proximity of chemotherapy and surgery to blood sampling. Tumour specimens available for 19 of the patients were additionally assayed for *ras* mutations and compared with blood specimens. Our results indicate mutant K-*ras* DNA is readily detectable by PCR in the plasma or serum of patients with advanced colorectal cancer. Thus, plasma- or serum-based nucleic acid amplification assays may provide a valuable method of monitoring and potentially detecting colorectal cancer.

**Keywords:** plasma; serum; K-*ras*; DNA; colorectal cancer; polymerase chain reaction

Colorectal cancer is a common and often fatal disease for which methods of early detection and monitoring are needed. While early detection and prompt medical intervention can be curative, many patients present with regional or widespread metastasis, reflecting in part the limitations of current screening methods. Mutations of the K-*ras* oncogene are detected in approximately 40% of patients with colorectal cancer, with these mutations occurring in the later stages of adenoma development and persisting during clonal transformation (Bos et al, 1987; Forrester et al, 1987; Vogelstein et al, 1988). As most K-*ras* mutations are restricted to a few adjacent nucleotides of codons 12 and 13, nucleic acid amplification assays, such as the polymerase chain reaction (PCR), can be used to detect small numbers of these mutant *ras* oncogenes. The diagnostic potential of this tumour-specific approach was demonstrated by Sidransky et al (1992), who used PCR to detect ras mutations in stool from patients with benign or malignant neoplasms of the colon. PCR has been used to detect K-*ras* mutations in the sputum of patients with lung cancer (Yakubovskaya et al, 1995) and in both pancreatic secretions and blood from patients with pancreatic cancer (Tada et al, 1993). Similarly, Hardingham et al (1995) demonstrated that circulating K-*ras* mutant colorectal cancer cells can be detected in blood using PCR. Nucleic acid amplification assays have also been used to detect circulating cancer cells in the blood of patients with various other malignancies, including lymphoma (Gribben et al, 1994), leukaemia (Pichert et al, 1994), prostate cancer (Moreno et al, 1992; Ghossein et al, 1995), malignant melanoma (Hoon et al, 1995; Smith et al, 1991), breast cancer (Datta et al, 1994) and hepatocellular cancer (Komeda et al, 1995).

*Received 4 November 1996*
*Revised 14 April 1997*
*Accepted 1 May 1997*

*Correspondence to:* CD Gocke, Department of Pathology, PO Box 850, The Pennsylvania College University School of Medicine, Milton S Hershey Medical Center, Hershey, PA 17033, USA

Most PCR-based studies evaluating the blood of cancer patients have focused on the cellular fraction of blood as they attempt to detect circulating cancer cells. An alternative and perhaps more efficacious method to detect mutant K-*ras* DNA involves analysis of the plasma or serum fraction of blood. Extracellular DNA normally circulates in the plasma component of blood in small amounts (Fournie et al, 1986), and significant increases in the amount of circulating DNA are noted in patients with cancer (Leon et al, 1977), possibly correlating with tumour viability (Stroun et al, 1987, 1989). Shapiro et al (1983) demonstrated that patients with gastrointestinal cancers have significant elevations of serum DNA compared with patients with benign gastrointestinal diseases. Recently, it has been shown that mutated *ras* oncogene DNA can be detected in plasma or serum by PCR. Sorenson et al (1994) found mutated K-*ras* DNA in the plasma of three patients with metastatic pancreatic cancer, with the sequences of mutated genes detected in plasma matching those of the primary tumour. Vasioukhin et al (1994) detected N-*ras* mutations in the plasma of patients with acute myelogenous leukaemia and myelodysplastic syndrome. Vasyukhin et al (1994) have similarly detected mutant K-*ras* DNA in the plasma from 6 of 15 patients with colorectal cancer.

In this larger study, we combine efficient DNA extraction methods with a sensitive PCR assay to detect mutant K-*ras* DNA in plasma and serum. We confirm that mutant K-*ras* DNA is readily detectable in the blood of patients with colorectal cancer, but not in the blood of normal volunteers. These findings suggest that analysis of blood plasma or serum for mutant oncogene DNA might provide a valuable method of monitoring and potentially diagnosing patients with colorectal cancer.

### MATERIALS AND METHODS

### Specimens

Five to ten millilitres of peripheral blood were collected from 31 patients with metastatic (30 patients) or unresected primary (one patient) colorectal cancer and from 28 normal volunteers. For plasma preparation, EDTA-coated or citrate Vacutainer tubes were used to avoid the potential inhibitory effects of heparin on the amplification assay (Beutler et al, 1990). In other cases, clotted blood specimens were obtained. Blood was centrifuged at 4°C and 850 ***g*** for 10 min, then the plasma (22 patients, 28 normal volunteers) or serum (nine patients) fraction was removed and stored frozen at – 70°C. Of the patient samples, 15 were collected prospectively and were stored for 1–6 months before assaying. The remaining 16 samples had been collected in 1988 and had been stored for more than 6 years before assaying. All normal volunteer samples had been collected recently and had been frozen for a short time. Paraffin blocks of tumours were available for 19 patients, including 18 primary tumours and four metastatic lymph node specimens.

### Plasma and serum DNA extraction

DNA was extracted from plasma or serum by one of two methods. In extraction method one (25 patients, 13 normal volunteers), adapted from Fedorov et al (1986), 200 µl of serum or plasma was mixed with an equal volume of 3.45 M sodium chloride, then boiled for 4 min, slowly cooled to room temperature over 30 min and refrigerated at 4°C for 20 h. The samples were then centrifuged at 3000 ***g*** for 30 min. One hundred and eighty microlitres of supernatant was removed and loaded in two separate aliquots onto a Sephadex G50 column (Quick Spin, Boehringer Mannheim, Indianapolis, IN, USA) equilibrated with TE (10 mM Tris pH 7.0, 1 mM EDTA) that had been prepared previously according to the manufacturer's instructions. The column was eluted at 1100 ***g*** for 4 min per aliquot. The resultant eluate was reduced in volume to approximately 70 µl by vacuum desiccation. One half of this amount was used in the PCR assay.

In extraction method two (six patients, 15 normal volunteers), plasma or serum DNA was co-precipitated with gelatin by a method modified from that of Fournie et al (1986). A stock 5% (w/v) gelatin solution was prepared by mixing 1 g of gelatin (G8-500, Fisher, Pittsburgh, PA, USA) with 20 µl of sterile, double-distilled water, autoclaving for 30 min and filtering through a 0.2-µm filter. The resultant solution was sequentially frozen in a dry ice/ethanol bath and thawed at room temperature for a total of five cycles. A working 0.3% gelatin solution was prepared by heating the stock solution to 60°C and mixing 600 µl of 5% gelatin with 25 µl of 1 M Tris-HCl (pH 8.0) and 9.4 µl of sterile, double-distilled water. One hundred and sixty microlitres of plasma or serum was mixed with 12.8 µl of 0.5 M EDTA and 467 µl of sterile, double-distilled water, then emulsified for 3 min with 320 µl of phenol. The solution was centrifuged at 14 000 ***g*** for 10 min, and 570 µl of the aqueous layer was removed to a clean tube. DNA was precipitated by addition of 142 µl of the 0.3% (w/v) gelatin working solution and 500 µl of cold absolute ethanol, followed by incubation at – 20°C for a minimum of 2 h. The samples were microfuged at 4°C for 15 min, washed once with cold 70% ethanol and dried in a 60°C heat block for 10 min. DNA was resuspended by the addition of 70 µl of sterile, double-distilled water preheated to 60°C. One-half of each sample was used in the PCR assay.

### PCR assay

Mutant K-*ras* oncogene DNA was isolated using a non-radioactive PCR assay adapted from Kahn et al (1991). PCR was performed as follows: a reaction mixture containing 35 µl of the DNA solution extracted from plasma or serum, 50 mM potassium chloride, 10 mM Tris pH 9.0, 0.1% Triton X-100, 1.5 mM magnesium chloride, 200 µM of each dATP, dGTP, dCTP and dTTP, 0.5 pmol oligonucleotide K-*ras*-L (5′-ACTGAATATAAACTTGTGGTA-GTTGGACCT-3′), 0.75 pmol oligonucleotide K-*ras*-R (5′-TCAAAGAATGGTCCTGGACC-3′) and 1 unit of *Taq* DNA polymerase (Promega, Madison, WI, USA) in a volume of 50 µl was prepared. The stated ratio of primers was found empirically to provide the cleanest results (not shown). The oligonucleotide K-*ras*-L is immediately upstream of codon 12 and is modified at the 28th base (G > C) to create an artificial restriction enzyme site (*Bst*NI). The oligonucleotide K-*ras*-R is modified at the 17th nucleotide (C > G) to create an artificial *Bst*NI site to serve as an internal control for completion of digestion. This approach differs from the original (Kahn et al, 1991), in which an unmodified K-*ras*-R primer was used in the first round and the modified primer only in the second round of amplification. Our approach is simpler and yields equivalent sensitivity (data not shown).

The reaction mixture was overlaid with mineral oil and cycled 15 or 20 times at 94°C for 48 s, 56°C for 90 s and 72°C for 155 s in a PHC-2 thermocycler (Techne, Princeton, NJ, USA). Ten microlitres of the PCR mixture were then removed to a new tube, adjusted to be equivalent to 1 × *Bst*NI reaction buffer, and 10 units of *Bst*NI restriction enzyme (Stratagene, La Jolla, CA, USA) were added and then incubated at 60°C for 90 min. A second aliquot of 10 units of BstNI was added and the reaction was continued for 90 min more. Ten microlitres of the digested PCR mixture was then removed to a new tube and a new reaction mixture was set up for the second amplification stage using identical constituents as in the first amplification, except that 7.7 pmol of oligonucleotide K-*ras*-L and 11.5 pmol of oligonucleotide K-*ras*-R were used. The same cycling conditions were used for 33 or 35 cycles. A second *Bst*NI restriction digestion was performed using 25 µl of the second-step PCR product and 17 units of enzyme in a final volume of 35 µl. Digestions were performed for 60 min, and then a second aliquot of 10 units of enzyme was added and digested for an additional 60 min. The final digestion product was electrophoresed through a 3% agarose gel.

All amplification assays included a mutated K-*ras*-positive control consisting of the colon carcinoma cell line GEO (codon 12 K-*ras* mutation GGT > GCT), a wild-type K-*ras* (mutant negative) control consisting of DNA from normal placenta tissue and a negative control lacking DNA. In addition, reactions were run in parallel without *Bst*NI digestion to ensure amplification had occurred. Routine precautions to prevent contamination were used in all amplification-based work (Kwok and Higuchi, 1989). The risk of contamination yielding falsely positive results was further minimized by repeating PCR assays on all patient plasma or serum samples two or three times on different days.

### Paraffin tissue extraction

DNA was harvested from tissue in available paraffin blocks by cutting 2–4 paraffin sections at a thickness of 15 microns using ethanol-cleaned microtome blades. The tissue was transferred to microfuge tubes and extracted twice with 300 µl of xylene to

© Cancer Research Campaign 1997

remove paraffin, then washed twice with 300 μl of 70% ethanol and finally with 300 μl of 10 mM Tris, 1 mM EDTA. The tissue was then resuspended in 300–500 μl of proteinase K buffer (200 mM Tris HCl pH 8.0, 100 mM EDTA, 1% sodium dodecyl sulphate, 500 μg ml$^{-1}$ proteinase K) and digested at 48°C for 24 to 48 h, with 100 μg of proteinase K added after 12 h of digestion. Serial extractions with equal volumes of phenol–chloroform and chloroform–isoamyl alcohol were performed, followed by precipitation with two volumes of ethanol and 300 mM sodium acetate, pH 5.6. The pellet was washed in 70% ethanol, dried and resuspended at 55°C for 10 min in 200 μl of 10 mM Tris, 1 mM EDTA. Two hundred microlitres of the DNA solution was loaded on a Sephadex G50 spin column in two aliquots and centrifuged according to the manufacturer's instructions. This was then reduced in volume to approximately 70 μl by vacuum desiccation.

PCR assays for K-*ras* mutations were performed essentially as described, with 35 μl of DNA solution added to the PCR reaction mixture. The two-stage PCR assay consisted of an initial 15-cycle amplification, followed by reamplification of digested products for an additional 30 cycles, with the final product being electrophoresed through a 3% agarose gel. Positive controls (GEO cell line) and negative controls (placenta tissue and reagents without template DNA) were included in all amplification assays. In addition, equivalent amounts of tumour DNA were run in parallel without *Bst*NI digestion to ensure that amplification at the locus had occurred.

### Gel electrophoresis

Twenty-five microlitres of the final digestion product were electrophoresed through a 3% agarose (2:1 NuSieve GTG, FMC Bioproducts, Rockland, ME, USA; Molecular Biology Grade Agarose, Promega) gel in 1 × TBE at 75 V DC for approximately 2 h before staining with ethidium bromide. Photographs of the gels were taken on an ultraviolet light transilluminator (Foto-prep, Fotodyne, Hartland, WI, USA). Mutant K-*ras* DNA was evident on the gel as a single band of length 142 bp, failure of digestion was evident as a band at 157 bp and cleaved non-mutated (wild-type) K-*ras* DNA was sometimes evident as a band at 113 bp.

### Sequence analysis

To confirm results of the plasma and serum specimen assays mutant DNA bands were excised from the agarose gel, reamplified, cloned into the pGEM-T vector plasmid (Promega) and sequenced using a commercial kit (Sequenase 2.0, USB, Cleveland, OH, USA). A minimum of two clones were sequenced for each PCR product, and all gave consistent results.

### Clinical correlations

Clinical variables that might affect plasma or serum K-*ras* detection were reviewed retrospectively. Comparisons were made between detection of mutant K-*ras* DNA in plasma or serum and patient sex, primary tumour location (colon vs rectum), principal metastatic site (liver vs lung) and proximity of chemotherapy or surgical resection to specimen blood draw. Statistical analysis was performed using Fisher's exact test for a two by two table (two-tail).

**Figure 1** Gel electrophoresis demonstrating detection of mutant K-*ras* DNA in plasma from colorectal cancer patients (lanes 4–7). Lane 1, uncut DNA control; lane 2, GEO cell line positive control; lane 3, 1:10 000 dilution of positive control; lane 8, placenta (wild-type) negative control; lane 9, DNA-absent negative control confirming lack of contamination; lane 10, molecular weight markers (Phi X174 DNA cut with *Hae*III)

### RESULTS

PCR was performed on blood plasma and serum, primary tumours and lymph node metastases. Serial dilutions indicate the sensitivity of the PCR assay to be one mutant gene out of $10^5$ normal genes (data not shown).

### Plasma and serum specimens

All 28 plasma and serum specimens from normal volunteers tested negative for mutant K-*ras* DNA regardless of extraction method used. Of 31 cancer patient plasma and serum specimens, 12 (39%) were positive for mutant K-*ras* DNA, with a distinct band seen on the gels at the 142 bp length (Figure 1). Positive samples included 8 of the 22 plasma specimens and four of the nine serum specimens. Both extraction methods appeared efficacious, with mutant K-*ras* detected in 8 of 25 specimens for which extraction method one was used and in four of six specimens for which extraction method two was used. All positive specimens were repeatedly positive on retesting of the specimen. The remaining 19 patient plasma and serum specimens repeatedly tested negative. To make certain that negative results were not due to failed amplifications, specimens were further tested by omitting the initial *Bst*NI digestion. The expected wild-type K-*ras* band was formed in all cases indicating that amplifiable DNA was present in each sample. In all PCR runs, the positive GEO control tested positive, and the negative placenta and DNA absent controls tested negative. The one patient without evidence of metastatic disease whose blood was drawn before surgical resection of his rectal primary tested negative for mutant K-*ras* DNA in his blood. His tumour was unavailable for testing.

### Tumour specimens

Tissue blocks were available for 19 patients, including 18 patients for whom the primary tumour specimen was available. One patient had only a metastatic nodal specimen available. Additional lymph node blocks were available in three other patients to compare with primary tumour specimens.

K-*ras* mutations were detectable in the tumours or nodes of six (32%) of these patients. Tumours from the remaining 13 patients

© Cancer Research Campaign 1997

**Table 1** Mutation sequences and tissue comparisons for patients with mutant K-*ras*-positive plasma or serum

| Patient | Blood specimen | | Tissue specimen |
|---|---|---|---|
| | Type of specimen | Mutation sequence | Mutant K-*ras* (+/–) |
| 1 | P | GAT | NA |
| 2 | S | GCT | – |
| 3 | S | GCT | + |
| 4 | P | GAT | NA |
| 5 | P | GAT | + |
| 6 | S | GAT | + |
| 7 | S | GAT | – |
| 8 | P | GAT | – |
| 9 | P | GAT | + |
| 10 | P | GAT | + |
| 11 | P | GTT,GAT | NA |
| 12 | P | GAT | NA |

P, plasma; S, serum; NA, tissue not available.

**Figure 2** Sequencing results from extracellular mutant K-*ras* DNA. A single representative clone from each of three patients is shown; two to four mutant clones were obtained from all patients. The mutant base in codon 12 is indicated on the left. The wild-type sequence reads (from bottom up) ACCTGGTGGC, with codon 12 underlined and the base altered by primer mismatching italicized (see text)

were negative for mutant K-*ras*. Plasma and serum specimens were correlated with the available tissue. Of the 19 patients, plasma or serum was positive for mutant K-*ras* DNA in eight patients, with tumours or nodes being mutant *ras* positive in five of these patients. Of the 13 patients with primary tumours negative for mutant K-*ras* DNA, plasma or serum was negative for *ras* mutations in ten. Thus, K-*ras* mutations were detectable in the blood but not in the tumours of three patients (Table 1, patients 2, 7 and 8). Only a single block of tumour was available for testing from each of these patients. In contrast, of the six patients who demonstrated K-*ras* mutations in their cancers, plasma or serum was positive for mutant *ras* DNA in five cases, with one patient having a *ras*-positive tumour and *ras*-negative blood. The available metastatic lymph node from that patient was similarly negative for *ras* mutation. With this exception, concordance for *ras* mutation was noted between primary tumours and available lymph nodes.

### Sequence analysis

Sequence analysis of plasma and serum PCR products from the 12 mutant *ras*-positive blood specimens confirmed that the detected K-*ras* DNA was mutated (Figure 2 and Table 1). These mutations included nine instances of GGT > GAT, two cases of GGT > GCT and one case of GGT > GTT plus GAT. Of note, three patients had mutant *ras* detected in their blood but not in their tumour. Two of these patients had GGT > GAT mutations, making contamination by the GEO control cell line, which carries a GCT mutation, unlikely. The third patient had a GGT > GCT mutation.

### Clinical correlations

All patients (18 men, 13 women) had either metastatic colorectal carcinoma (30 patients) or an unresected but not metastatic primary rectal cancer (one patient) at the time of their blood draw, with parenchymal metastasis involving predominantly liver (18 patients), lung (five patients), both liver and lung (five patients) or omentum/ascites (two patients). Initial primary tumours originated from the colon in 20 patients and from the rectum in 11 patients. Mutant K-*ras* DNA could be detected in patient plasma or serum regardless of patient sex, initial primary tumour location (colon vs rectum) or principal parenchymal metastatic site (liver vs lung). Differences in the detection of mutant *ras* DNA among patients grouped by these clinical variables were not significant, although group sizes were small (Table 2).

The proximity of specimen blood draw to chemotherapy and surgery were further investigated. While recognizing that advanced colorectal cancer responds poorly to chemotherapy, it is possible that chemotherapy might increase circulating mutant *ras* through cellular necrosis or apoptosis or might decrease circulating mutant

 © Cancer Research Campaign 1997

**Table 2** Correlation between clinical variables and detection of mutant K-*ras* DNA in plasma or serum

| Clinical variable | No. of patients | No. of patients with mutant K-*ras* DNA in blood | *P*-value |
|---|---|---|---|
| Sex | | | |
| Male | 18 | 8 | 0.48 |
| Female | 13 | 4 | |
| Primary tumour | | | |
| Colon | 20 | 10 | 0.13 |
| Rectum | 11 | 2 | |
| Principal metastatic site | | | |
| Liver | 18 | 9 | 1.00 |
| Lung | 5 | 2 | |
| Chemotherapy | | | |
| Within 3 months | 13 | 5 | 1.00 |
| Naive or free > 6 months | 13 | 6 | |

*ras* by a reduction in tumour burden. Chemotherapeutic histories were available for 29 patients. Of these, 13 patients had a history of having received chemotherapy within 3 months before their specimen blood draw, with mutant K-*ras* DNA detected in the plasma or serum of five of these patients, including three of the seven patients who had received chemotherapy within the preceding month. However, 13 other patients had their blood sample drawn before initiation of chemotherapy, i.e. while still chemotherapy-naive (nine patients), or after having been chemotherapy-free for at least 6 months (four patients). Mutant K-*ras* DNA was detected in the plasma or serum in six of these patients (four chemotherapy-naive patients, two chemotherapy-free patients). Differences regarding circulating mutant *ras* DNA in the recent chemotherapy and chemotherapy-naive/-free groups were not significant ($P = 1.00$). Similarly, surgery might release circulating tumour cells or traumatize cells, promoting mutant *ras* detection. However, of the 12 patients with K-*ras* mutations detected in their blood, only one patient had the blood specimen drawn within 1 month after surgery, while seven had their blood specimen drawn more than 1 year after surgery. Conclusions cannot be extrapolated to the intraoperative and immediate post-operative periods, as no blood specimens were obtained during these times. Thus, the above data demonstrate that mutant K-*ras* DNA circulates in plasma or serum even without recent surgical trauma or chemotherapy-induced necrosis or apoptosis.

Blood specimens from 16 patients were collected in 1988 and stored frozen. Mutations were detected in the plasma or serum in four of these specimens, indicating that mutant K-*ras* DNA can be detected in plasma and serum frozen long term. Whether the sensitivity of the assay is diminished when specimens are stored under these conditions remains uncertain.

### DISCUSSION

Mutation of the K-*ras* oncogene is recognized as an early and frequent mutational event in the pathogenesis of colorectal cancer, making it a potentially valuable clinical marker. As the location of *ras* point mutations is fairly restricted, with most occurring at codons 12, 13 or 61, they are particularly amenable to detection by nucleic acid amplification assays (Bos, 1989). In this study we have combined DNA extraction methods with a non-radioactive PCR assay to demonstrate that mutant K-*ras* DNA is readily detectable in the plasma and serum of patients with advanced colorectal cancer. The presence of mutant ras DNA in plasma has similarly been demonstrated by others. Sorenson et al (1994) combined a dialysis-based DNA extraction procedure with PCR to detect mutant K-*ras* DNA in the plasma of patients with pancreatic cancer. Vasioukhin and associates (1994) used PCR to amplify DNA extracted from plasma through centrifugation on a caesium sulphate gradient and were able to demonstrate circulating N-*ras* mutations in patients with leukaemia and myelodysplastic syndrome. Vasyukhin et al (1994) similarly extracted DNA from the plasma of patients with colorectal cancer and were able to amplify mutant K-*ras* DNA. While we used different DNA extraction methods, sensitivity and reproducibility were maintained. DNA extraction method two in particular has the advantage of being rapid, with DNA extracted from plasma in as little as 3 h.

Although we did not demonstrate complete concordance between K-*ras* mutations in plasma or serum and those in tumours, it appears that we were able to detect most cases with circulating mutant K-*ras* DNA. Our detection of circulating mutant ras in 39% of the patients is similar to what would be expected based upon the incidence of K-*ras* mutations in colorectal cancer. There was a single case of a primary tumour bearing a K-*ras* mutation that was not detectable in blood. Of interest, examination of a metastatic node from the patient also failed to detect mutant ras, raising the possibility that the patient's remaining metastatic tumours either lost or never carried the mutation. Three patients had K-*ras* mutations detectable in their blood but not in their primary tumours. Results for these three patients probably indicate circulating extracellular mutant K-*ras* DNA, with several factors making contamination unlikely: all normal control specimens tested negative; placenta and DNA-absent controls tested negative; results were consistently reproducible upon retesting of the specimens; and sequence analysis confirmed specific mutations, of which two of the three cases differed from the positive control. Other investigators have reported *ras* mutations in the metastasis but not in the corresponding primary tumour, or in the primary tumour but not in the metastasis (Oudejans et al, 1991). Sampling of blood plasma or serum might prove a particularly efficacious means of detecting mutant DNA should such tumour heterogeneity be present, because it effectively screens the entire tumour burden.

It is not known whether mutant K-*ras* DNA in plasma and serum represents extracellular DNA released from viable tumour, DNA

© Cancer Research Campaign 1997

released from necrotic or apoptotic cells or DNA released from the lysis of fragile circulating cancer cells. It is well documented that circulating cancer cells can be detected in blood (Hardingham et al, 1995). However, it has been observed both by us and by others (Vasioukhin et al, 1994; Vasyukhin et al, 1994) that mutant *ras* can be detected in plasma even when not detected in the cellular fraction of blood, suggesting that circulating cancer cells may not be involved. Cellular necrosis and apoptosis are common processes in cancer. Conceivably, such processes could yield detectable amounts of extracellular DNA, either as free DNA or as membrane-bound apoptotic bodies. An alternative possibility is that detectable extracellular DNA is being shed from viable tumour (Leon et al 1977). An active release of DNA that appears to be independent of cell death but under regulatory control has been demonstrated in lymphocytes (Rogers et al, 1972; Anker et al, 1975). Furthermore, DNA has been demonstrated on the cell surface of malignant cell lines (Aggarwal et al, 1975; Juckett and Rosenberg, 1982). It is commonly assumed that shed extracellular DNA would be susceptible to digestion by plasma DNAases. However, Leon et al (1981) found inhibitors of DNAase in the plasma of cancer patients, which might lead to accumulation of circulating DNA. Others have suggested that DNA is shed in a protein or proteolipid complex and is thus resistant to degradation (Rogers et al, 1974; Stroun et al, 1977, 1987).

Regardless of the origin of this extracellular DNA, the use of plasma-based nucleic acid amplification assays to detect tumour-specific extracellular DNA represents a new and potentially valuable approach to cancer detection and monitoring. This initial study was limited primarily to patients with metastatic disease, and the use of our assay in detecting early disease remains undefined. In general, the applicability of K-*ras* assays to cancer detection and monitoring will be limited by the incidence of K-*ras* mutations, with over half of colorectal cancers reported to be mutant K-*ras* negative. However, the concept of selective amplification of mutant DNA from plasma or serum can be extended to other oncogenes and tumour-suppressor genes, including *p53*, which is frequently mutated in colorectal cancer. Although the mutated nucleotides in the *p53* gene are not as clustered as in the K-*ras* oncogene, a panel or multiplex approach using a number of primers could allow increased detection of cancers. Furthermore, it has recently been demonstrated that tumour-associated microsatellite alterations can be detected in the plasma DNA of small-cell lung cancer patients (Chen et al, 1996) and the serum DNA of head and neck cancer patients (Nawroz et al, 1996). As microsatellite alterations are often present in colorectal tumours (Lothe et al, 1993; Bocker et al, 1996), they should similarly be detectable in the plasma and serum DNA of colorectal patients. Plasma and serum-based assays aimed at cancer monitoring might additionally be improved using a quantitative PCR approach. The screening, diagnostic and monitoring potential of detecting oncogenes or other tumour-associated DNA in plasma and serum and the pathophysiological function and clinical implication of their presence warrant further investigation.

### ACKNOWLEDGEMENTS

We gratefully acknowledge Mrs Sharon Lewis for her secretarial assistance and thank Dr Vernon Chinchilli for the statistical analysis. We also thank Drs K Mulder and A Buard for their gift of the GEO cell line.

### REFERENCES

Aggarwal SK, Wagner RW, McAllister PK and Rosenberg B (1975) Cell-surface-associated nucleic acid in tumorigenic cells made visible with platinume–pyrimidine complexes by electron microscopy. *Proc Natl Acad Sci USA* **72**: 928–932

Anker P, Stroun M and Maurice PA (1975) Spontaneous release of DNA by human blood lymphocytes as shown in an in vitro system. *Cancer Res* **35**: 2375–2382

Beutler E, Gelbart T and Kuhl W (1990) Interference of heparin with the polymerase chain reaction. *Biotechniques* **9**: 166

Bocker T, Schlegel J, Kullmann F, Stumm G, Zirngibl H, Epplen JT and Ruschoff J (1996) Genomic instability in colorectal carcinomas: comparison of different evaluation methods and their biological significance. *J Pathol* **179**: 15–19

Bos JL (1989) Ras oncogenes in human cancer: A review. *Cancer Res* **49**: 4682–4689

Bos JL, Fearon ER, Hamilton SR, Vries, MV, van Boom JH, van der Eb AJ and Vogelstein B (1987) Prevalence of ras gene mutations in human colorectal cancers. *Nature* **327**: 293–297

Chen XQ, Stroun M, Magnenat JL, Nicod LP, Kurt AM, Lyautey J, Lederrey C and Anker P (1996) Microsatellite alterations in plasma DNA of small cell lung cancer patients. *Nature Med* **2**: 1033–1035

Datta YH, Adams PT, Drobyski WR, Ethier S, Terry VH and Roth MS (1994) Sensitive detection of occult breast cancer by the reverse-transcriptase polymerase chain reaction. *J Clin Oncol* **12**: 475–482

Fedorov NA, Yaneva IS, Skotnikova OI and Pan'kov VN (1986) DNA assay in human blood plasma. *Bull Exp Biol Med* **102**: 1190–1192

Forrester K, Almonguera C, Han K, Grizzle, WE and Perucho M (1987) Detection of high incidence of K-ras oncogenes during human colon tumorigenesis. *Nature* **327**: 298–303

Fournie GJ, Gayral-Taminh M, Bouche J-P and Conte JJ (1986) Recovery of nanogram quantities of DNA from plasma and quantitative measurement using labelling by nick translation. *Anal Biochem* **158**: 250–256

Ghossein RA, Scher HI, Gerald WI, Kelly WK, Curley T, Amsterdam A, Zhang Z-F and Rosai J (1995) Detection of circulating tumor cells in patients with localized and metastatic prostatic carcinoma: clinical implications. *J Clin Oncol* **13**: 1195–1200

Gribben JG, Neuberg D, Barber M, Moore J, Pesek KW, Freedman AS and Nadler LM (1994) Detection of residual lymphoma cells by polymerase chain reaction in peripheral blood is significantly less predictive for relapse than detection in bone marrow. *Blood* **83**: 3800–3807

Hardingham JE, Kotasek D, Sage RE, Eaton MC, Pascoe VH and Dobrovic A (1995) Detection of circulating tumor cells in colorectal cancer by immunobead-PCR is a sensitive prognostic marker for relapse of disease. *Mol Med* **1**: 789–794

Hoon DSB, Wang Y, Dale PS, Conrad AJ, Schmid P, Garrison D, Kuo C, Foshag LJ, Nizze AJ and Morton DL (1995) Detection of occult melanoma cells in blood with a multiple-marker polymerase chain reaction assay. *J Clin Oncol* **13**: 2109–2116

Juckett DA and Rosenberg B (1982) Actions of cis-diamminedichloroplatinum on cell surface nucleic acids in cancer cells as determined by cell electrophoresis techniques. *Cancer Res* **42**: 3565–3573

Kahn SM, Jiang W, Culbertson TA, Weinstein IB, Williams GM, Tomita N and Ronai Z (1991) Rapid and sensitive nonradioactive detection of mutant K-*ras* genes via 'enriched' PCR amplification. *Oncogene* **6**: 1079–1083

Komeda T, Fukuda Y, Sando T, Kita R, Furukawa M, Nishida N, Amenomori M and Nakao K (1995) Sensitive detection of circulating hepatocellular carcinoma cells in peripheral venous blood. *Cancer* **75**: 2214–2219

Kwok S and Higuchi R (1989) Avoiding false positives with PCR. *Nature* **339**: 237–238

Leon SA, Shapiro B, Sklaroff DM and Yaros MJ (1977) Free DNA in the serum of cancer patients and the effect of therapy. *Cancer Res* **37**: 646–650

Leon SA, Shapiro B, Servi P and Parsons RG (1981) A comparison of DNA and DNA-binding protein levels in malignant disease. *Eur J Cancer* **17**: 533–538

Lothe RA, Peltomäki P, Meling GI, Aaltonen LA, Nyström-Lahti M, Pylkkänen L, Heimdal K, Andersen TI, Moller P, Rognum T, Fossa SD, Haldorsen T, Langmark F, Brogger A, de la Chapelle A and Borresen A-L (1993) Genomic instability in colorectal cancer: relationship to clinicopathological variables and family history. *Cancer Res* **53**: 5849–5852

Moreno JG, Croce CM, Fischer R, Monne M, Vihko P, Mulholland SG and Gomella LG (1992) Detection of hematogenous micrometastasis in patients with prostate cancer. *Cancer Res* **52**: 6110–6112

Nawroz H, Koch W, Anker P, Stroun M and Sidransky D (1996) Microsatellite alterations in serum DNA of head and neck cancer patients. *Nature Med* **2**: 1035–1037

© Cancer Research Campaign 1997

Oudejans JJ, Slebos RJC, Zoetmulder FAN, Moor WJ and Rodenhuis S (1991) Differential activation of *ras* genes by point mutation in human colon cancer with metastases to either lung or liver. *Int J Cancer* **49**: 875–879

Pichert G, Alyea EP, Soiffer RJ, Roy D-C and Ritz J (1994) Persistence of myeloid progenitor cells expressing BCR-ABL mRNA after allogeneic bone marrow transplantation for chronic myelogenous leukemia. *Blood* **84**: 2109–2114

Rogers JC, Boldt D, Kornfeld S, Skinner A and Valeri CR (1972) Excretion of deoxyribonucleic acid by lymphocytes stimulated with phytohemagglutinin or antigen. *Proc Natl Acad Sci USA* **69**: 1685–1689

Shapiro B, Chakrabarty M, Cohn EM and Leon SA (1983) Determination of circulating DNA levels in patients with benign or malignant gastrointestinal disease. *Cancer* **51**: 2116–2120

Sidransky D, Tokino T, Hamilton SR, Kinzler KW, Levin B, Frost P and Vogelstein B (1992) Identification of *ras* oncogene mutations in the stool of patients with curable colorectal tumors. *Science* **256**: 102–105

Smith B, Selby P, Southgate J, Pittman K, Bradley C and Blair GE (1991) Detection of melanoma cells in peripheral blood by means of reverse transcriptase and polymerase chain reaction. *Lancet* **338**: 1227–1229

Sorenson GD, Pribish DM, Valone FH, Memoli VA, Bzik DJ and Yao S-L (1994) Soluble normal and mutated DNA sequences from single-copy genes in human blood. *Cancer Epidemiol Biomarkers Prev* **3**: 67–71

Stroun M, Anker P, Maurice PA and Gahan PB (1977) Circulating nucleic acids in higher organisms. *Int Rev Cytol* **51**: 1–48

Stroun M, Anker P, Lyautey J, Lederrey C and Maurice PA (1987) Isolation and characterization of DNA from the plasma of cancer patients. *Eur J Cancer Clin Oncol* **23**: 707–712

Stroun M, Anker P, Maurice P, Lyautey J, Lederrey C and Beljanski M (1989) Neoplastic characteristics of the DNA found in the plasma of cancer patients. *Oncology* **46**: 318–322

Tada M, Omata M, Kawai S, Saisho H, Ohto M, Saiki R and Sninsky JJ (1993) Detection of *ras* gene mutations in pancreatic juice and peripheral blood of patients with pancreatic adenocarcinoma. *Cancer Res* **53**: 2472–2474

Vasioukhin V, Anker P, Maurice P, Lyautey J, Lederrey C and Stroun M (1994) Point mutations of the N-*ras* gene in the blood plasma DNA of patients with myelodysplastic syndrome or acute myelogenous leukaemia. *Br J Haematol* **86**: 774–779

Vasyukhin V, Stroun M, Maurice P, Lyautey J, Lederrey C and Anker P (1994) K-*ras* point mutations in the blood plasma DNA of patients with colorectal tumors. In *Challenges of Modern Medicine*, Volume 5, *Biotechnology Today*, Verna R and Shamoo A (eds) pp. 141–150. Area-Serono Symposia Publications: Rome

Vogelstein B, Fearon ER, Hamilton SR, Kern SE, Preisinger AC, Leppert M, Nakamura Y, White R, Smits AMM and Bos JL (1988) Genetic alterations during colorectal-tumor development. *N Engl J Med* **319**: 525–532

Yakubovskaya MS, Spiegelman V, Luo FC, Malaev S, Salnev A, Zborovskaya I, Gasparyan A, Polotsky B, Machaladze Z, Trachtenberg AC, Belitsky GA and Ronai Z (1995) High frequency of K-*ras* mutations in normal appearing lung tissues and sputum of patients with lung cancer. *Int J Cancer* **63**: 810–814

© Cancer Research Campaign 1997